# Seasonal body mass dynamics mediate life‐history trade‐offs in a hibernating mammal

**DOI:** 10.1111/1365-2656.70160

**Published:** 2025-10-18

**Authors:** Austin Z. T. Allison, Courtney J. Conway, Amanda R. Goldberg, Alice E. Morris, Emma C. Hakanson

**Affiliations:** ^1^ Idaho Cooperative Fish and Wildlife Research Unit, Department of Fish and Wildlife Sciences University of Idaho Moscow Idaho USA; ^2^ U.S. Geological Survey, Idaho Cooperative Fish and Wildlife Research Unit University of Idaho Moscow Idaho USA; ^3^ Present address: Department of Biology Colorado State University 1878 Campus Delivery Fort Collins Colorado 80523 USA; ^4^ Present address: Western Ecological Research Center, U.S. Geological Survey San Diego Field Station‐Santa Ana Office 1801 East Chestnut Avenue Santa Ana California 92701 USA

**Keywords:** costs of reproduction, food limitation, foraging behaviour, predation avoidance, resource acquisition and allocation, seasonal environments, state‐dependent life histories, survival

## Abstract

1. Energetic acquisition and growth are key traits that affect demography and life‐history strategies. Many animals that live in seasonal environments in which food availability fluctuates store energy endogenously as fat in anticipation of food shortage. Fat‐storing mammalian hibernators are an extreme example of this strategy wherein the optimal resolution of resource allocation trade‐offs is essential to survival. Hence, these species provide an opportunity to test potential causes and consequences of seasonal body mass dynamics.
2. We used a 12‐year dataset of 8753 body mass records from 3351 individually marked northern Idaho ground squirrels (*Urocitellus brunneus*)—a federally threatened hibernator—to meet three objectives: (1) document seasonal body mass changes by sex, age and reproductive status, (2) test ecological hypotheses to explain spatiotemporal variation in body mass and (3) document fitness consequences of pre‐hibernation body condition via condition‐dependent overwinter survival.
3. Squirrels varied substantially in seasonal body mass dynamics. The magnitude (36–155%) and onset (late May to early July) of rapid active‐season mass gain varied among demographic groups. Reproductive females acquired the necessary fat stores to survive hibernation later in the active season than did males and non‐reproductive females. Moreover, squirrels with better pre‐hibernation body condition were more likely to survive to the subsequent year, potentially because they allocated excess energetic reserves to prolonging hibernation via early immergence and thereby reduced predation risk. These results suggest a direct trade‐off between current and future reproduction mediated by resource acquisition and allocation, as predicted by life‐history theory.
4. Colder active‐season temperatures and lower conspecific densities negatively influenced squirrel body condition, possibly via reductions in foraging activity associated with those conditions. These ecological effects on body condition constrain resource allocation and demographic outcomes. As such, our results can help guide research and conservation strategies to benefit hibernating animals.

## Graphical abstract

We tested a suite of ecological hypotheses to explain variation in seasonal body mass dynamics of a fat‐storing mammalian hibernator. We further demonstrated that pre‐hibernation mass gain in ground squirrels mediates an annual allocation trade‐off between current and future reproduction as the squirrels forage and rear young under predation risk.

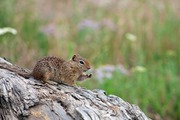

## 1 INTRODUCTION

Energy acquisition and growth are key traits that affect demography and life‐history strategies. Animals often evolve state‐dependent life‐history strategies because food availability and/or endogenous energetic reserves can strongly influence the optimal resolution of allocation trade‐offs (McNamara & Houston, 1996). Individuals can solve allocation dilemmas through greater resource acquisition, which reduces the severity of life‐history trade‐offs by increasing fecundity and longevity (van Noordwijk & de Jong, 1986). Because allocation trade‐offs influence demographic vital rates and population dynamics, food limitation is often invoked as a primary ecological determinant of animal abundance (White, 2008). Indeed, supplemental food often increases fecundity and/or survival, and hence, population density (Boutin, 1990). However, food acquisition is not the only task animals must optimize. Competition, thermal tolerance and predation can negatively influence energetic state by imposing costs on access to food (Brown, 1988; Lima, 1998; Long et al., 2014). As such, identifying the ultimate drivers of growth and body condition and the demographic consequences thereof can provide insight into life‐history evolution and population dynamics and help generate more effective conservation strategies for animals threatened with extinction.

Food abundance fluctuates seasonally in many systems, and seasonal accumulation and depletion of endogenous energetic reserves in response to those fluctuations are common phenomena in animals. Herbivores in temperate regions often increase body condition across spring and summer, when food is readily available, and spend accrued capital during fall and winter, when food is scarce or of low quality (Hodges et al., 2006; Lima, 1986; Parker et al., 2009). Fat‐storing mammalian hibernators arguably represent the most exaggerated expression of this pattern. These animals accumulate fat stores in white adipose tissue during the active season, which is subsequently metabolized while animals fast (sometimes for many months) during hibernation (MacCannell et al., 2017). Individuals of some hibernating species increase their fat mass by several fold, resulting in a total body mass increase of >100% during their annual active season (Davis, 1976; Humphries et al., 2003; Sheriff et al., 2013). Moreover, some hibernators allocate excess energetic reserves—beyond those required to survive the annual period of food shortage—to prolonging hibernation via earlier onset of seasonal heterothermy (Allison et al., 2023; Bieber et al., 2014). This strategy makes sense because hibernation confers significant survival benefits due to reduced predation during dormancy (Turbill et al., 2011). These adaptations to food seasonality under predation risk demonstrate the potential influence of endogenous energetic state on the evolution of animal life histories by suggesting a mechanistic link between seasonal body mass dynamics and demography. Consequently, fat‐storing hibernators are a system well‐suited to elucidating the causes and consequences of intraspecific variation in body mass.

We sought to better understand the seasonal body mass dynamics of a fat‐storing mammalian hibernator threatened with extinction: the northern Idaho ground squirrel (*Urocitellus brunneus*). The species is listed as federally threatened under the US Endangered Species Act (US Fish and Wildlife Service, 2000), and <2500 individuals persist in a fragmented metapopulation within a small area in western Idaho, USA (Wagner & Jolly, 2025). These small‐bodied, semi‐fossorial squirrels undergo one of the longest documented annual hibernation periods of any mammal: 8 to 10 months per year (approximately July–April), and age, sex, body condition and environmental conditions explain some of the intraspecific variation in hibernation duration (Allison et al., 2023). Squirrels in good condition in early summer prolong hibernation by up to 1 month via early immergence compared to squirrels in poor condition (Allison et al., 2023). In doing so, these individuals may markedly reduce predation risk and thereby increase the probability of surviving to reproduce in the subsequent year (Allison, Conway, & Goldberg, 2024). The lengthy annual hibernation period leaves squirrels only a few months during which they reproduce and attain the necessary fat stores to again hibernate until the subsequent spring. Reproductive male squirrels (≥2‐years‐old) emerge from hibernation ~2 weeks before females to initiate spermatogenesis in anticipation of the breeding season (Allison et al., 2023). Females ≥1‐year‐old mate within a few days of emergence from hibernation, give birth ~23 days later, and wean pups after ~30 days of nursing (males do not provide parental care; Sherman, 1989; Yensen & Sherman, 1997). Both sexes rapidly gain mass after their reproductive roles conclude for the year and enter hibernation in mid‐summer. Juveniles grow rapidly and subsequently fatten after emerging from natal burrows and enter hibernation ~3 weeks after adults (Allison et al., 2023). The squirrels exhibit considerable variation in the timing and magnitude of seasonal mass gain among and within populations and years.

Little is known about the ecological mechanisms underpinning intraspecific variation in northern Idaho ground squirrel body mass or the demographic repercussions of this variation despite the importance of both hibernation and seasonal mass dynamics to the life history of these imperilled squirrels. Nevertheless, several hypotheses posit food limitation as a likely cause of apparent past population declines in this rare species. Hence, filling this knowledge gap can yield important conservation implications alongside a greater understanding of the interplay between seasonal mass dynamics and life‐history evolution. Potential causes of food limitation in the squirrels include forage quality and quantity (Sherman & Runge, 2002), competition with larger, congeneric Columbian ground squirrels (*Urocitellus columbianus*; Allison, Conway, & Goldberg, 2024; Yensen & Dyni, 2020), effects of thermal conditions on foraging profitability (Allison & Conway, 2022) and reduced foraging activity under predation risk associated with a lack of shared vigilance at low population densities (Allison & Conway, 2022). If food limitation regulates northern Idaho ground squirrel population dynamics, we might expect one or more of the above mechanisms to exert control over seasonal body mass dynamics. Specifically, food availability per se, active‐season temperature and conspecific density (assuming facilitation) are predicted to positively influence squirrel body mass under the respective hypotheses, and competitor density (conspecific and congeneric) is predicted to negatively influence body mass if competition limits availability of or access to food. Moreover, seasonal body mass dynamics may be dependent on the timing of emergence from hibernation. Later‐emerging squirrels might be lighter on a given day because they potentially use more energy during hibernation and have less time to replenish endogenous stores after emergence. This leads to the prediction that snowmelt timing, which is closely tied to hibernation emergence in northern Idaho ground squirrels (Allison, Conway, Morris, et al., 2024), negatively influences date‐adjusted squirrel body mass. We aimed to test predictions of life‐history theory and to provide a stronger mechanistic foundation for future conservation efforts by fulfilling three objectives related to seasonal body mass dynamics in northern Idaho ground squirrels: (1) document seasonal mass changes by sex, age class and reproductive status, (2) test the above ecological hypotheses to explain spatiotemporal variation in body mass (Table 1) and (3) document overwinter survival consequences of pre‐hibernation body condition.

**TABLE 1:** Hypotheses to explain spatiotemporal variation in northern Idaho ground squirrel body mass, predictions we used to test the hypotheses, and the underlying ecological mechanisms and assumptions linking the hypotheses and predictions.

Hypothesis	Ecological mechanisms	Key assumptions	Predictions tested	Support?
Hibernation emergence Timing	Time limitation	Snowmelt timing positively influences hibernation emergence timing, which limits annual foraging time prior to a given date (Allison et al., 2023; Allison, Conway, Morris, et al., 2024)	Snowmelt timing is negatively correlated with body mass	Partial
Forage availability	Food limitation	Lower food abundance and/or quality reduces foraging efficiency as squirrels expend more time/energy to acquire the same absolute amount of food (Sherman & Runge, 2002)	Active‐season NDVI (a proxy for food availability) is positively correlated with body mass	No
Thermal conditions	Thermal tolerance	Squirrels spend more time in burrows and less time foraging and/or store less fat per unit of food consumed when ambient temperatures are colder (Allison & Conway, 2022)	Active‐season temperature is positively correlated with body mass	Yes
Conspecific density	Intraspecific competition or facilitation	Intraspecific competition for food reduces foraging efficiency by reducing food availability or accessibility (Allison & Conway, 2022; Allison, Conway, & Goldberg, 2024); alternatively, conspecific density facilitates foraging activity through enhanced vigilance (Allison & Conway, 2022)	Conspecific density is negatively correlated with body mass (competition); alternatively, conspecific density is positively correlated with body mass (facilitation)	Yes (Facilitation)
Competitor density	Interspecific competition	Interspecific competition for food with congeneric Columbian ground squirrels reduces foraging efficiency by reducing food availability or accessibility (Allison, Conway, & Goldberg, 2024; Yensen & Dyni, 2020)	Columbian ground squirrel density is negatively correlated with body mass	No

*Note*: We found support for the thermal conditions and conspecific density hypotheses. We found partial support for the hibernation emergence timing hypothesis in that mean snowmelt date at study sites negatively influenced squirrel body mass but interannual variation in snowmelt timing did not. We found no support for the forage availability and competitor density hypotheses.

## 2 MATERIALS AND METHODS

### 2.1 Study system

We conducted this study across 12 years (2013–2024) at 16 study sites scattered throughout the ~1600‐km^2^ distribution of the northern Idaho ground squirrel. The study sites (1230–1700 m above sea level) also covered much of the species' elevational range. Cold, snowy winters and warm, dry summers characterized the study area, but the rugged topography of the region caused significant climatic variation among study sites (Table S1). Interannual weather variation further contributed to the environmental variation experienced by squirrels in our study (Table S1). Forbs and grasses—the primary food of northern Idaho ground squirrels—greened up shortly after spring snowmelt, flowered and fruited into early summer, and senesced by late summer (Goldberg, Conway, Tank, et al., 2020). Some squirrels moved from the open areas where they lived during the active season to surrounding forests and woodlands to hibernate (Goldberg, Conway, Evans Mack, & Burak, 2020). Columbian ground squirrels coexisted with the smaller northern Idaho ground squirrels at 13 of the 16 study sites. The larger squirrels burrowed in woodlands, along forest‐meadow ecotones, and in patches of deeper soil within meadows (Allison, Conway, & Goldberg, 2024; Yensen & Dyni, 2020). Many predators—including American badgers (*Taxidea taxus*), coyotes (*Canis latrans*), long‐tailed weasels (*Mustela frenata*) and red‐tailed hawks (*Buteo jamaicensis*)—hunted ground squirrels during the active season (Yensen & Sherman, 1997), but only badgers excavated and consumed hibernating squirrels (Morris, 2025).

### 2.2 Data collection

We captured free‐ranging northern Idaho ground squirrels and Columbian ground squirrels each year from 2013 to 2024, although we did not trap at each study site in each year (range = 4–12 consecutive years per site). Study sites varied from 4 to 8 ha in area and were separated from the nearest neighbour site by 140 to 6700 m (often including significant topography and/or other barriers to squirrel movement). We trapped at each site on 8 to 12 discontinuous days per year from April to August to document seasonal changes in body mass within each site. We placed traps (Tomahawk Live Trap, Hazelhurst, WI, USA) at squirrel burrows throughout a given study site on each trapping day. We trapped for 6 to 10 h per day, depending on weather.

We marked all squirrels with ear tags (National Tag & Band Co., Newport, KY, USA) and/or a passive integrated transponder (PIT) tag (Biomark, Boise, ID, USA) to allow for individual identification upon recapture. We recorded the sex and age class (juvenile or ≥1‐year‐old) of each squirrel. For northern Idaho ground squirrels, we later separated ≥1‐year‐olds into two age classes (yearlings and adults ≥2‐years‐old) for our analyses because previous research suggested that yearlings weigh less than older squirrels (Sherman & Runge, 2002). We designated unknown‐age squirrels (first captured at ≥1‐year‐old) as yearlings in the first year they were captured because long‐distance dispersal is rare in northern Idaho ground squirrels (Sherman & Runge, 2002), and we assumed we marked most resident squirrels as juveniles or yearlings given that annual capture probability was >0.9 in most years of the study (Allison, Conway, & Goldberg, 2024). We recorded the reproductive status (lactating or not) of yearling and adult female northern Idaho ground squirrels. We weighed each northern Idaho ground squirrel to the nearest gram. In total, we amassed 8753 body mass records from 3351 northern Idaho ground squirrels.

Our intensive annual trapping and marking effort provided the data to calculate a site‐specific annual index of adult population density for both species of ground squirrel (number of ≥1‐year‐old squirrels captured/trapping plot area). These density estimates allowed us to examine whether higher squirrel densities led to reduced body mass (via intra‐ and/or interspecific competition) or increased body mass (via shared vigilance that allowed more foraging time).

We downloaded three site‐level environmental datasets from which we created predictor variables for use in models of squirrel body mass: (1) 1‐km^2^‐resolution Snow Data Assimilation System (SNODAS) daily snow depth estimates (National Operational Hydrologic Remote Sensing Center, 2004), (2) 4‐km^2^‐resolution gridMET daily maximum temperature estimates (Abatzoglou, 2013) and (3) 30‐m^2^‐resolution Landsat weekly (every 8 days) normalized difference vegetation index (NDVI) data (Masek et al., 2006; Vermote et al., 2016). We generated three annual metrics for each study site from these data: (1) snowmelt date (last day on or after 1 Mar with snow depth ≥10 cm), (2) mean daily maximum temperature during the ground squirrel active season (1 May–31 July) and (3) mean NDVI during the active season (1 May–31 July). Snowmelt date—a proxy for hibernation emergence timing (Allison, Conway, Morris, et al., 2024)—allowed us to test the hypothesis that squirrels that emerge later from hibernation weigh less on a given day of the year because they have less time to forage and gain mass prior to that day. Active‐season temperature allowed us to test the hypothesis that colder temperatures negatively influence squirrel body mass through a reduction in foraging profitability related to thermal energetics. Active‐season NDVI—a proxy for food availability for generalist herbivores, such as ground squirrels (Pettorelli et al., 2005; Tamian et al., 2024)—allowed us to test the hypothesis that the abundance or quality of food constrains ground squirrel body condition.

We conducted the research described in this manuscript with permission from the University of Idaho Institutional Animal Care and Use Committee (protocols #2015‐53, #2019‐28, and #2022‐10). The US Fish and Wildlife Service (recovery permit #TE94776A) and Idaho Department of Fish and Game (scientific collecting permits #120629, #37330, and #65730) further permitted our research.

### 2.3 Statistical analyses

We used intrinsic and environmental variables to estimate seasonal body mass curves for northern Idaho ground squirrels and to test ecological hypotheses to explain spatiotemporal variation in body mass. We conducted all statistical analyses in R v4.4.2 (R Core Team, 2024). Snowmelt date and active‐season temperature were collinear (*r* = −0.62). Consequently, we used within‐group (study site) centring to decompose those variables into their spatial (among‐site variation in mean values) and temporal (within‐site interannual variation relative to the site mean) components (van de Pol & Wright, 2009). Mean snowmelt date and mean active‐season temperature were collinear with study site elevation (snowmelt: *r* = 0.87, temperature: *r* = −0.80) and each other (*r* = −0.89). Hence, we compared three potential global models by Akaike's information criterion corrected for small sample size (AIC_c_; Burnham & Anderson, 2002): one each that included a fixed effect of elevation, mean snowmelt date and mean active‐season temperature. We proceeded with the model that included mean snowmelt date, which produced the lowest AIC_c_ value (ΔAIC_c_ = 9.01 for the model containing mean temperature and ΔAIC_c_ = 11.38 for the model containing elevation).

We modelled squirrel body mass as a Gaussian distribution via linear mixed‐effects models in the lme4 package (Bates et al., 2015). The global model included fixed effects of mean snowmelt date, relative snowmelt date, relative active‐season temperature, active‐season NDVI, conspecific density, Columbian ground squirrel density and a three‐way interaction among day of year (third‐degree polynomial), squirrel sex and squirrel age class. The third‐degree polynomial and the three‐way interaction tested for nonlinear mass gain during the active season that reflects differential optimal life‐history strategies among demographic groups. The model also included crossed random intercepts for individual squirrel ID, study site and year. We compared all subsets of the global model by AIC_c_, and we present results from the top model to contain no uninformative predictor variables (85% confidence intervals overlap 0; Arnold, 2010; Sutherland et al., 2023; Table S2).

We used a subset of body mass data from reproductive‐age (yearling and adult) female squirrels to test for an effect of reproductive effort on seasonal mass dynamics. We created a binary predictor variable based on our reproductive status data from female squirrels captured and weighed between 15 May and 1 July in a given year, when females nursed pups. We designated squirrels recorded as lactating at any point during the nursing period as reproductive (742 squirrel‐years) and squirrels never recorded as lactating during that period as non‐reproductive (444 squirrel‐years). We modelled body mass of these females (3452 body mass records from 768 squirrels) via a linear mixed‐effects model with the same structure as the top model of our full body mass dataset, except for the effect of squirrel sex (which was not relevant to this model). We compared that informed null model by AIC_c_ to a model that additionally included a two‐way interaction between day of year (third‐degree polynomial) and our binary reproduction variable to test for an effect of reproduction on seasonal mass dynamics.

Lastly, we used our estimated seasonal body mass curves to produce a pre‐hibernation body condition metric as a predictor variable for use in survival models. We compared empirical body mass records to model‐based predictions from our top model of the full body mass dataset by dividing a recorded squirrel mass by the predicted mass for a squirrel of that sex and age class on that day of the year at that site. This resulted in a metric where values >1 denoted squirrels in relatively good condition (heavier than expected) and values <1 denoted squirrels in relatively poor condition (lighter than expected). When possible, we averaged the two summer body condition values most temporally proximate to 1 July for yearlings and adults and 1 August for juveniles. We chose these dates as the point immediately before squirrels of a given age class began to enter hibernation in our study area (juveniles initiated hibernation later than older squirrels) to maximize the predictive power of body condition on hibernation immergence timing and hence on overwinter survival probability. We retained single body condition estimates for use in survival models when we only captured a squirrel once in a given summer. Averaging two values, when possible, minimized the impact of daily body mass fluctuations on our results. We only considered yearling and adult squirrels captured after 1 June and juvenile squirrels captured after 1 July in a given year to avoid the inclusion of body mass records too temporally distant (i.e. >1 month) from hibernation decision‐making to be of high predictive value.

We modelled overwinter recapture probability (the probability that a squirrel captured in the summer of year *t* was recaptured in year *t* + 1) as a means of estimating the effect of pre‐hibernation body condition on overwinter survival (survival from summer to the subsequent spring breeding season). Modelling recapture rates did not allow us to account for imperfect detection of living squirrels in this analysis, nor did the procedure account for emigration out of the trapping area (even short‐distance dispersal events could result in some squirrels permanently leaving the trapping area). However, standard capture–mark–recapture survival models (e.g. Cormack–Jolly–Seber models) rigidly require that individual time‐varying covariates—like body condition—be measured or imputed for all individuals at each capture occasion in the analysis (Lebreton et al., 1992; White & Burnham, 1999). Using outdated body condition values or imputing values for uncaptured and possibly deceased squirrels is clearly inappropriate because body condition is a plastic trait that fluctuates across time and has no meaning for deceased individuals. We were confident that recapture probability largely reflected survival due to the high interannual site fidelity of northern Idaho ground squirrels (Sherman & Runge, 2002) and previously estimated high annual capture probabilities in our study system (>0.9; Allison, Conway, & Goldberg, 2024). We modelled recapture probability as a binomial distribution via a logit link function within generalized linear mixed‐effects models we constructed in the lme4 package. Our global model (based on 2467 overwinter return intervals for 1856 squirrels) included additive fixed effects of squirrel sex, age class and pre‐hibernation body condition, and crossed random intercepts for individual squirrel ID, study site and year. We compared all subsets of the global model by AIC_c_, and we present results from the top model that did not contain any uninformative predictor variables (Table S3).

## 3 RESULTS

**FIGURE 1:**
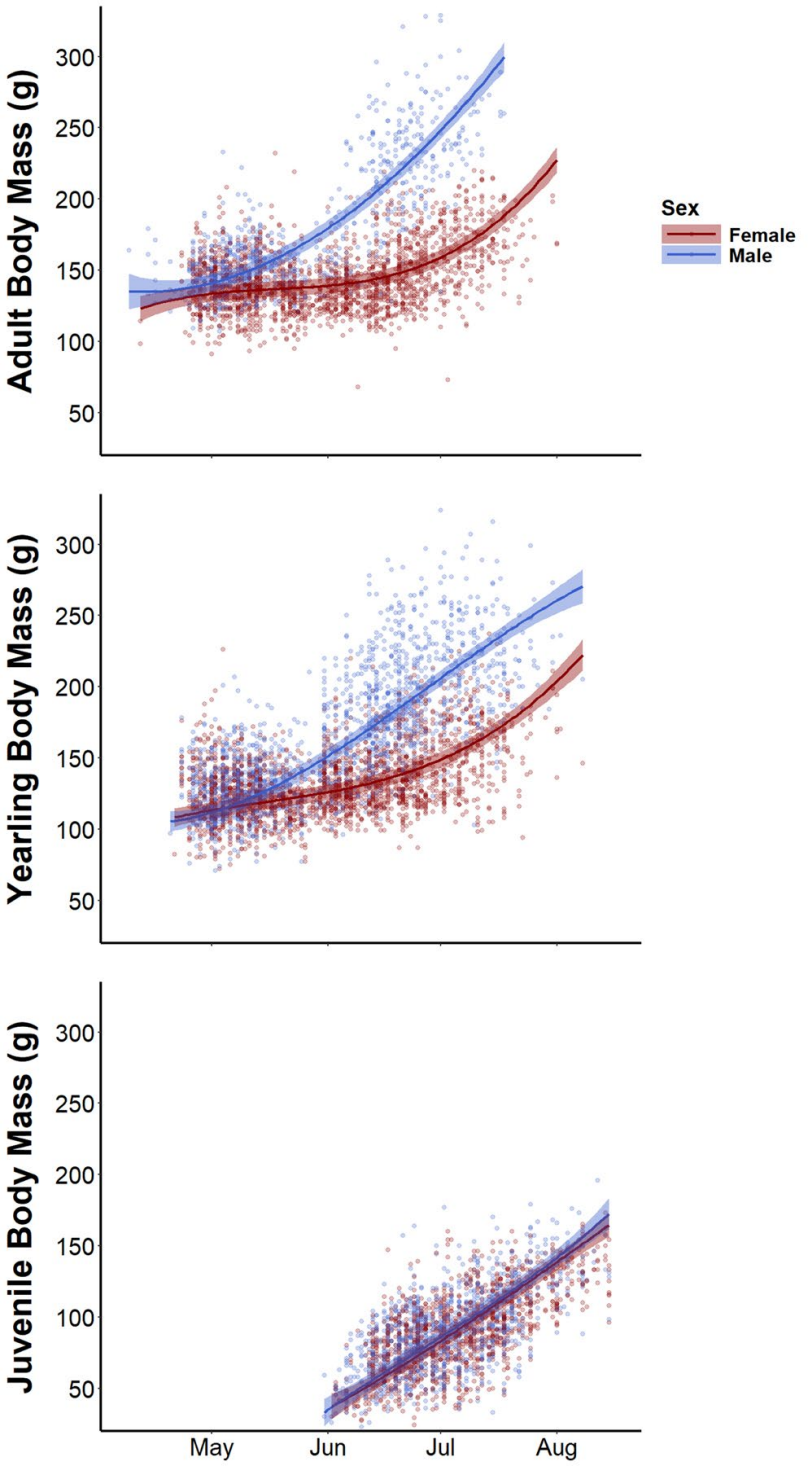
Seasonal body mass curves (±95% CI) for northern Idaho ground squirrels generated from the top linear mixed‐effects model to explain intraspecific variation in squirrel body mass based on 8753 body mass records from 3351 squirrels. All age and sex classes gained mass across the active season in anticipation of hibernation. Yearling and adult males rapidly gained mass earlier in the year and attained greater absolute pre‐hibernation body masses than females in those age classes. Juvenile body masses were similar between the sexes throughout the active season, and juveniles gained mass in a more linear fashion than older age classes. Adult squirrels weighed more than yearlings, which in turn weighed more than juveniles. Points represent raw mass records.

Only one model of northern Idaho ground squirrel body mass receiving ≥0.01 Akaike model weight (*w*_*i*_)—the top model—contained no uninformative predictor variables (the other potentially competitive models performed worse by adding uninformative variables to the structure of the top model; Tables S2 and S4–S7). Several intrinsic and environmental variables influenced northern Idaho ground squirrel body mass (Table S4). The fixed effects in the top model of our complete squirrel body mass dataset explained 75% of intraspecific variation in body mass; the full model (including random effects) explained 88% of intraspecific variation. The interactive effects of day of year, sex and age class explained much of that variation. Squirrels of all age and sex classes gained substantial mass during the active season, but the magnitude and timing of mass gain varied substantially among demographic groups (Figure 1, Table S4). Mass gain accelerated in yearlings and adults as the active season progressed but proceeded in a more linear fashion in juveniles. Yearling and adult squirrels weighed 36%–104% more on 15 July than on 1 May (at the reference site, holding environmental variables at their mean), depending on their sex and age. Juveniles of both sexes weighed ~155% more on 1 August than on 15 June. Yearling and adult males were heavier than yearling and adult females, respectively, but differences in the timing and magnitude of active‐season mass gain meant that the disparity between the sexes within these age classes was minimal early in the year and peaked in mid‐summer. Adult females weighed 133.4 ± 2.8 g (estimate ± SE) on 1 May, while adult males weighed 141.0 ± 2.9 g (5% heavier). In comparison, adult females weighed 181.9 ± 3.0 g on 15 July, at which time adult males weighed 290.3 ± 4.6 g (60% heavier). Juvenile body mass was similar between the sexes throughout the active season after their emergence from natal burrows.

**FIGURE 2:**
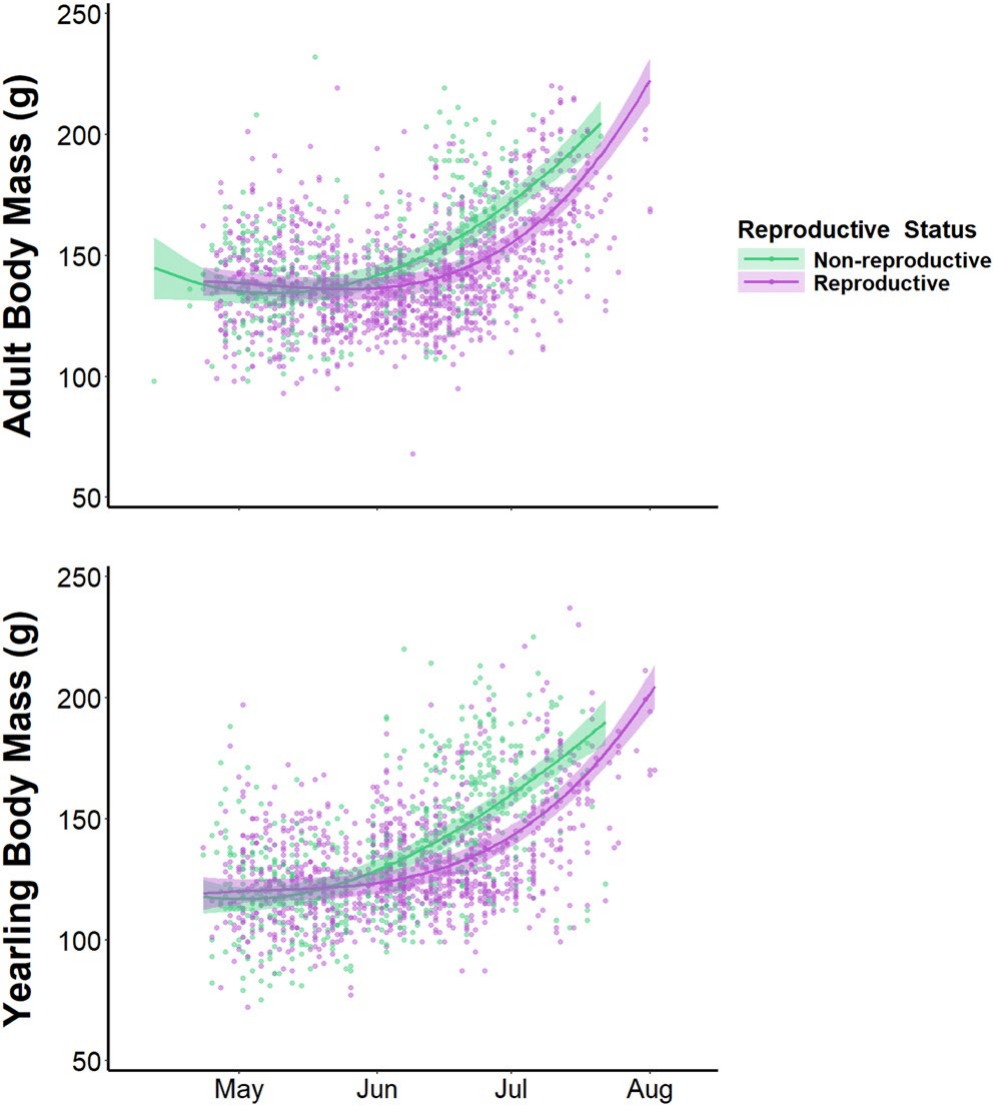
Seasonal body mass curves (±95% CI) for female northern Idaho ground squirrels (those for which we documented reproductive status) generated from the top linear mixed‐effects model to explain intraspecific variation in body mass based on 3452 body mass records from 768 squirrels. Non‐reproductive females exhibited earlier onset of rapid mass gain than reproductive females. As such, non‐reproductive females accumulated the necessary reserves to survive winter sooner than did reproductive females. Points represent raw body mass records.

The later onset of rapid pre‐hibernation mass gain in yearling and adult female squirrels compared to males appeared to result from differential reproductive requirements between the sexes. Our model of female body mass (based on the subset of females for which we documented reproductive status) that included a date‐dependent effect of reproduction performed significantly better than the informed null model (ΔAIC_c_ = 227.03), which indicated a strong effect of reproduction on seasonal mass gain. The fixed effects in the model explained 39% of intraspecific variation in body mass, and the full model explained 65% of intraspecific variation. Reproductive and non‐reproductive females had similar body masses on 1 May (reproductive: 138.5 ± 2.5 g, non‐reproductive: 135.3 ± 2.5 g, non‐reproductive females 3% lighter; Figure 2). However, non‐reproductive females began to rapidly gain mass earlier than reproductive females and weighed significantly more by 15 July (reproductive: 177.9 ± 2.5 g, non‐reproductive: 193.9 ± 3.5 g, non‐reproductive females 9% heavier). The timing of mass gain in non‐reproductive females matched that of male squirrels, but these females did not attain the pre‐hibernation body mass of males.

**FIGURE 3:**
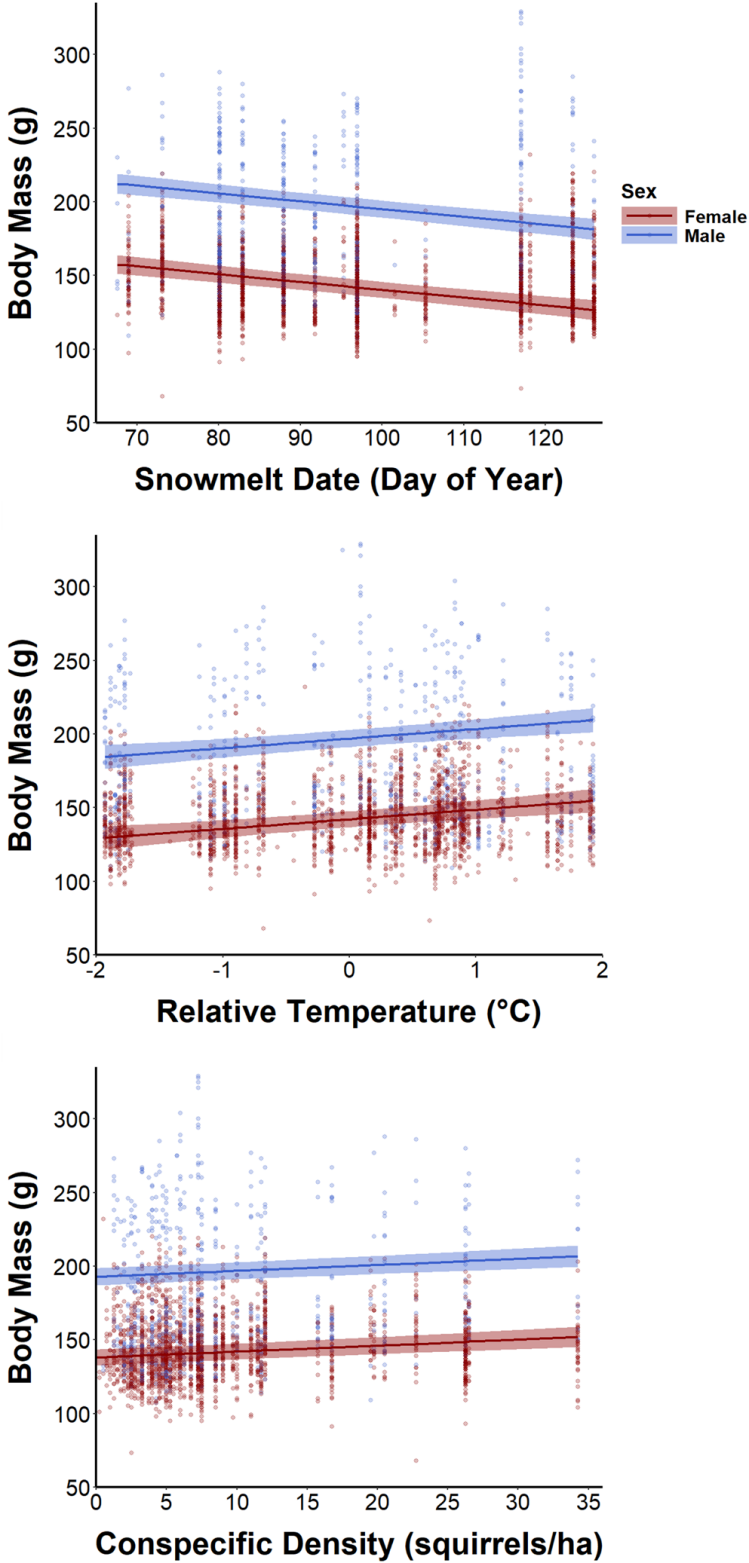
Marginal effects (±95% CI) of environmental variables on adult northern Idaho ground squirrel body mass generated from the top linear mixed‐effects model to explain intraspecific variation in squirrel body mass based on 8753 body mass records from 3351 squirrels. Mean snowmelt date at a site negatively influenced squirrel body mass. Annual active‐season temperature relative to the site mean positively influenced squirrel body mass. Conspecific density positively influenced squirrel body mass. Environmental variables similarly influenced yearling and juvenile body mass because the estimated effects were additive. Points represent raw body mass records.

Mean snowmelt date, active‐season temperature and conspecific density additively influenced northern Idaho ground squirrel body mass (Table S2). Conversely, we failed to detect effects of relative snowmelt date, NDVI and Columbian ground squirrel density (Tables S2 and S5–S7). Among‐site variation in mean snowmelt date negatively influenced squirrel body mass. With the day of year held at its mean (~10 June), adult females at a site with a mean snowmelt date of 9 March weighed 157.5 ± 3.3 g compared to 126.5 ± 3.5 g (25% lighter) for adult females at a site with a mean snowmelt date of 6 May (Figure 3, Table S4). Interannual variation in within‐site active‐season temperature positively influenced squirrel body mass. Adult females weighed 129.5 ± 3.9 g in years where active‐season temperatures were 1.9°C cooler than average at a given site compared to 154.6 ± 4.0 g (20% heavier) in years 1.9°C warmer than average. Conspecific density positively influenced squirrel body mass. Adult females at sites with a conspecific density of 1 squirrel/ha weighed 138.3 ± 2.8 g compared to 152.0 ± 3.5 g (10% heavier) at sites with a conspecific density of 34 squirrels/ha. However, relatively few squirrels experienced densities >12 squirrels/ha, a density at which adult females weighed 142.8 ± 2.7 g (4% heavier than at the most sparsely populated sites).

**FIGURE 4:**
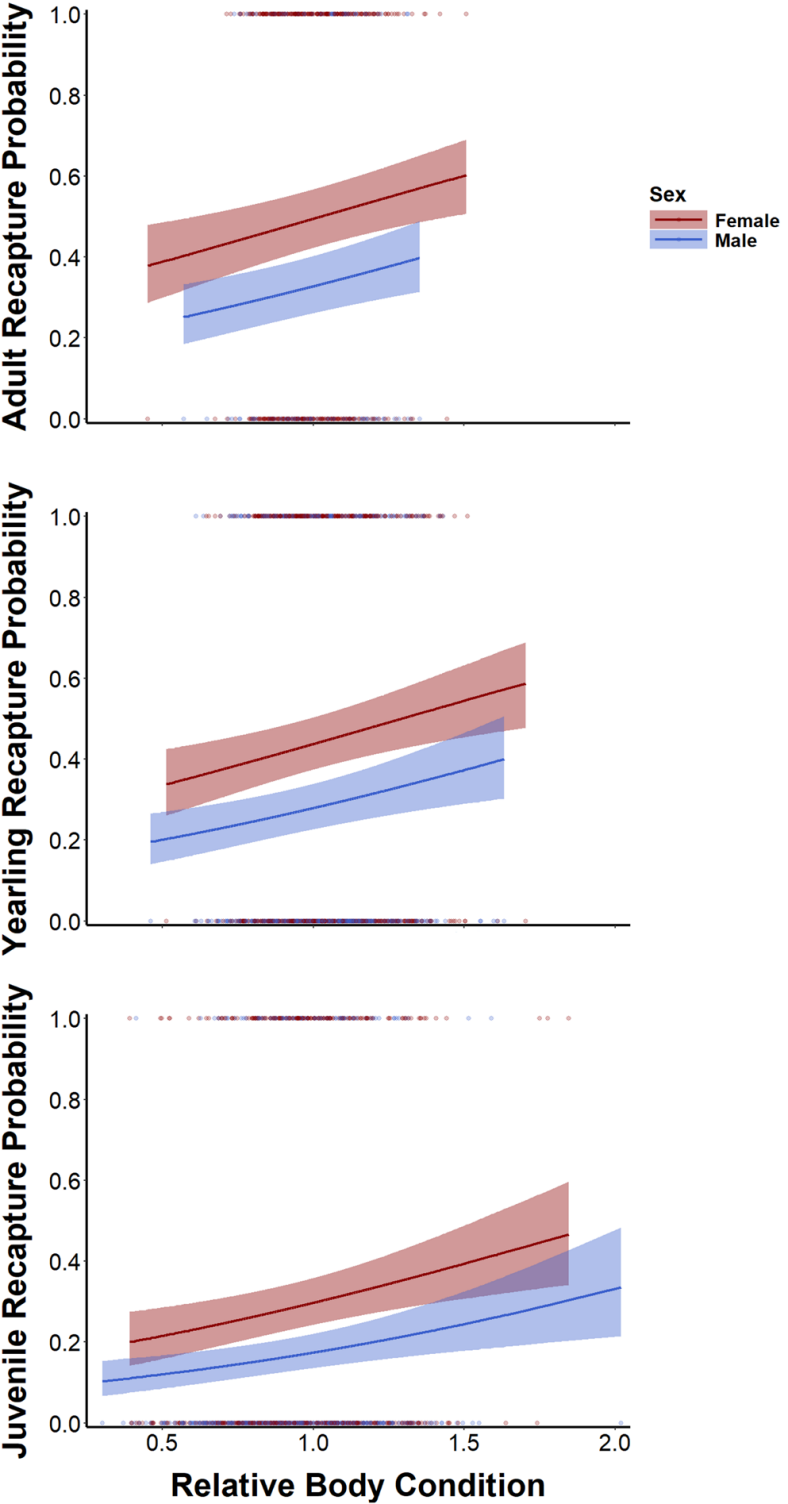
Marginal effects (±95% CI) of pre‐hibernation body condition on northern Idaho ground squirrel recapture probability (i.e. survival probability) generated from the top generalized linear mixed‐effects model to explain intraspecific variation in survival probability based on 2467 overwinter return intervals for 1856 squirrels. Pre‐hibernation body condition positively influenced overwinter survival probability of squirrels. Points represent raw recapture outcomes (0 = not recaptured, 1 = recaptured).

Squirrel sex, age class and pre‐hibernation body condition additively influenced northern Idaho ground squirrel overwinter survival probability (Table S2). The fixed effects in the top model explained 8% of intraspecific variation in recaptures, and the full model explained 15% of intraspecific variation. Squirrels in better condition in early summer were more likely to survive until the subsequent active season (Figure 4, Table S8). Despite the relatively small fraction of variation in survival probability explained by body condition, the estimated effect size was large enough to be of ecological significance. The ranges of estimated recapture probabilities across the full range of observed body condition values for each sex and age class were as follows: 0.38 ± 0.05 to 0.60 ± 0.05 (worst condition to best condition) for adult females, 0.25 ± 0.04 to 0.40 ± 0.05 for adult males, 0.34 ± 0.04 to 0.59 ± 0.05 for yearling females, 0.20 ± 0.03 to 0.40 ± 0.05 for yearling males, 0.20 ± 0.03 to 0.47 ± 0.07 for juvenile females and 0.10 ± 0.02 to 0.34 ± 0.07 for juvenile males. For a given body condition, females were more likely to survive overwinter than were males; adults were more likely to survive overwinter than were yearlings, which in turn were more likely to survive overwinter than were juveniles.

## 4 DISCUSSION

Seasonal environments impose numerous evolutionary pressures and constraints that often delineate trade‐offs that form the basis of animal life histories. However, the inherent predictability of seasonality allows animals to evolve life‐history strategies that anticipate future environmental conditions (McNamara & Houston, 2008). Seasonal variation in food availability poses a particularly strong challenge to animals for the obvious reason that animals require energy to carry out daily tasks and somatic processes that promote survival and reproduction. Consequently, animals have evolved a diversity of remarkable physiological and behavioural adaptations to avoid the worst potential negative consequences of seasonal energetic shortfall. Hibernation allows some mammals to remain in place through inhospitable seasonal conditions by reducing metabolic requirements during bouts of torpor to as little as 2% of their normothermic baseline (Heldmaier & Ruf, 1992). Yet, months‐long annual hibernation periods still require significant energetic preparation in the form of either food‐caching or endogenous fat‐storage (Davis, 1976; Humphries et al., 2003). We demonstrated that northern Idaho ground squirrels meet this annual challenge by depositing endogenous fat stores such that their body mass drastically increases during the active season, similar to many other hibernating sciurids (Armitage et al., 1976; Boswell et al., 1994; Buck & Barnes, 1999; Carrier et al., 2022; Dobson et al., 1992; Fagerstone, 1988; Schwanz, 2006). Indeed, the squirrels clearly acquired fat stores more than sufficient to survive the annual period of unprofitable foraging, as demonstrated by the fact that nearly all squirrels hibernated earlier than was necessary from an energetic perspective (i.e. before the onset of snowfall and cold temperatures while food is still relatively abundant in the form of seeds, roots and some late‐season green vegetation; Allison et al., 2023). Hibernating during periods of environmental suitability likely represents a predation avoidance strategy deployed by these small squirrels (Bieber & Ruf, 2009; Constant et al., 2025). Hence, the ability to accumulate substantial energetic reserves in a limited time is a vital aspect of the squirrels' life history and may be key to the species' persistence.

The contours of seasonal mass gain differed among sexes, age classes and reproductive status in a manner that exemplifies differences in optimal life‐history strategies among demographic groups. Differences in body mass dynamics between males and females within yearling and adult age classes likely reflect their annual reproductive cycles. Squirrels breed only during a short window as females emerge from hibernation, and males are subsequently freed from immediate reproductive considerations until the subsequent year (Sherman, 1989; Sherman & Runge, 2002). Intensive intrasexual competition for mates (Sherman, 1989) and shorter hibernation periods (Allison et al., 2023) apparently result in lower annual survival for males compared to females. However, reproductive considerations in males seemingly impose a comparatively small cost in terms of mass gain. Reproductive females gained mass slowly while nursing litters—the most energetically intensive life‐history stage for most female mammals (Speakman, 2008)—and only rapidly accumulated fat stores after weaning pups. In contrast, non‐reproductive females exhibited seasonal mass dynamics that more closely resembled those of males (i.e. relatively early rapid mass gain). Relatively early mass gain potentially allowed non‐reproductive females to enter hibernation earlier and thereby reduce predation risk and increase the probability of surviving to reproduce in the subsequent year (Allison et al., 2023). The faster mass gain by non‐reproductive females coupled with the positive effect of pre‐hibernation body condition on overwinter survival apparently reflected a classic allocation‐based trade‐off between current and future reproduction mediated by endogenous energetic reserves (Stearns, 1992; Williams, 1966). That is, lower survival as a result of delayed fat deposition and entrance into hibernation may be a direct cost of reproduction in female ground squirrels. We assume reproductive‐age females mate and rear young when feasible such that non‐reproductive females adopt an alternative life‐history tactic to make the best of a bad job (i.e. state‐dependent life histories; McNamara & Houston, 1996), but we cannot rule out the possibility that fixed alternative life‐history strategies coexist within the species (i.e. some yearlings may delay reproductive maturity in favour of increasing future reproductive capacity through additional growth; Gross, 1996). Further, some females that fail to wean litters may successfully mate and subsequently lose or abandon litters due to predation or poor environmental conditions early in the season.

The relatively linear mass gain exhibited by juvenile squirrels may have been caused by three related constraints: (1) Juveniles must grow structurally prior to hibernation to maximize future survival and reproductive success, (2) juveniles must attain sufficient fat stores to survive hibernation and (3) juveniles may have relatively little time to meet these needs. Previous research on hibernating mammals tended to emphasize the latter point and suggested that juvenile squirrels may be particularly vulnerable to environmental variation that influences the reproductive phenology of adults (Falvo et al., 2019; Howland et al., 2024; Lane et al., 2012; Lenihan & Van Vuren, 1996; Ozgul et al., 2010). If so, juvenile hibernators may be subject to a trade‐off between energetic allocation to structural growth and fat deposition, proxies for reproductive capacity and survival. However, little direct evidence supports the assumption that juvenile hibernators run up against seasonal energetic limitations that force the onset of hibernation. The linear growth we observed suggests that juveniles benefit from continuous rapid mass gain until they enter hibernation. Even so, juvenile northern Idaho ground squirrels in relatively good pre‐hibernation condition enter hibernation earlier than those in relatively poor condition, which indicates that the active season of juveniles is not energetically constrained in all cases (Allison et al., 2023). Further, we found no evidence that the relatively large amount of within‐site interannual variation in snowmelt timing during our study influenced squirrel body mass, as would be expected if squirrel mass gain and overwinter survival are strongly constrained by interannual variation in phenology. This contrasts with past research in hibernating sciurids (Carrier et al., 2022; Chmura et al., 2023; Ozgul et al., 2010) and with the effect of mean snowmelt date. It is possible that spatial differences in body mass associated with snowmelt timing instead reflect temperature, which was collinear, although this would contradict the positive interannual effect of temperature that we observed. Future work in this area may prove fruitful for the conservation of northern Idaho ground squirrels because relatively low overwinter survival of juveniles played a proximate role in past population declines (Sherman & Runge, 2002).

Effects of temperature on animal behaviour (Wong & Candolin, 2015) and demography (Selwood et al., 2015) have received significant attention in recent years as ecologists seek to document the consequences of anthropogenic climate change. Much of this research focused on the potential negative effects of heat stress (Pörtner & Farrell, 2008). Interestingly, then, squirrels apparently benefited from warmer active‐season temperatures in the form of increased body mass. A likely explanation for this pattern is that warmer temperatures experienced by squirrels in our study improved foraging conditions, which allowed squirrels to more efficiently accumulate energetic reserves (Allison & Conway, 2022). However, the benefits of increasing temperatures are limited as extreme heat and solar radiation reduce foraging activity in northern Idaho ground squirrels (Allison & Conway, 2022), and it remains unclear to what extent behavioural thermoregulation and foraging decisions can buffer animals against rapid climate warming (Cunningham et al., 2021).

Conspecific density positively influenced northern Idaho ground squirrel body mass—albeit mildly—an intriguing result given that intraspecific competition is often cited as a primary agent regulating population dynamics (Brook & Bradshaw, 2006) and a previous study found squirrel survival to be negatively density‐dependent (Allison, Conway, & Goldberg, 2024). Group‐living animals, such as northern Idaho ground squirrels, may benefit from the presence of conspecifics through shared vigilance and an associated increase in foraging activity (Alexander, 1974). These squirrels live in aggregations of 5–500 individuals (Wagner & Jolly, 2025) and spend more time foraging at higher conspecific densities, but whether predation risk or scramble competition for food causes this pattern is unknown (Allison & Conway, 2022). Possibly, competition and facilitation effects of conspecific density largely cancel each other out, resulting in the small positive effect size that we observed. The squirrels emit alarm calls in the presence of predators and regularly vocalize in their absence. It would be useful to know whether such vocalizations serve as a signal or cue of conspecific density or group vigilance used by individuals to modulate foraging effort, as are jump‐yips in black‐tailed prairie dogs (*Cynomys ludovicianus*; Hare et al., 2014). Positive effects of conspecific density can have important conservation implications for rare species because populations that fall below certain thresholds may be unable to recover on their own (Dennis, 1989; Stephens & Sutherland, 1999). Alternatively, the positive association between conspecific density and body mass that we documented may reflect latent environmental conditions that simultaneously promote population growth and body condition (e.g. food availability) and were not captured by variables included in our analyses (e.g. NDVI). Hence, the possibility of a positive effect of conspecific interactions on body mass in northern Idaho ground squirrels deserves additional attention as managers seek to recover the species.

The lack of support for effects of NDVI and Columbian ground squirrel density on northern Idaho ground squirrel body mass may also prove important from a conservation perspective. These negative results do not exclude common hypotheses to explain the rarity of the threatened squirrels, but they may provide information regarding the mechanisms underpinning the hypotheses. Sherman and Runge (2002) speculated that woody vegetation encroachment into meadows occupied by squirrels reduced soil moisture, and consequently, the quantity and quality of available forage. Our results contradict this hypothesis. However, woody vegetation encroachment resulting from fire suppression may cut off dispersal corridors or render former habitat unsuitable through alternative mechanisms (e.g. by altering predation risk). Likewise, prior studies suggested that Columbian ground squirrels compete with the smaller squirrels for food (Allison, Conway, & Goldberg, 2024; Yensen & Dyni, 2020). This would make sense as the two species have overlapping diets (Dyni & Yensen, 1996). Yet, we failed to demonstrate an effect of Columbian ground squirrel density on northern Idaho ground squirrel body mass. Columbian ground squirrels, though, may exclude the smaller squirrels from patches of shared preference, resulting in apparent niche partitioning (i.e. the ghost of competition) and little direct effect on body mass (Rosenzweig, 1991; Yensen & Dyni, 2020). Alternatively, northern Idaho ground squirrels may not be food limited per se, but instead time limited, as suggested by the effects of temperature and conspecific density on foraging activity and body mass. If so, the coexisting squirrels in our system may instead compete for some other resource (e.g. burrowing space; Allison, Conway, & Goldberg, 2024) or indirectly via apparent competition (Morris, 2025).

The demographic consequences of variation in ground squirrel body mass were clear: northern Idaho ground squirrels in better condition prior to the onset of the hibernation season were ~25% more likely to survive to the subsequent year compared to squirrels in poor condition. Even if most squirrels fall within a range of body condition values that results in a ~10% difference in overwinter survival probability, that could have important consequences for the population dynamics of these threatened squirrels. This result corroborates past work in hibernating mammals (Howland et al., 2024; Lenihan & Van Vuren, 1996; Murie & Boag, 1984; Ozgul et al., 2010; Schorr et al., 2009). Previous studies generally emphasized the limited time available for hibernators to fatten prior to hibernation, and so implied that many individuals fail to reach critical pre‐hibernation energetic storage thresholds. However, northern Idaho ground squirrels—like most hibernating species (Humphries et al., 2003)—apparently accumulate the necessary fat stores to survive the annual period of environmental unsuitability with relative ease. Therefore, we propose that the positive effect of pre‐hibernation body mass on overwinter survival that we observed—and possibly in many other systems—instead reflects state‐dependent maximization of hibernation duration as a means of avoiding predation (Allison et al., 2023). Some northern Idaho ground squirrels may indeed starve during hibernation in years with late spring snowmelt (Allison, Conway, Morris, et al., 2024), but even these mortalities may represent indirect costs of predation rather than poor pre‐hibernation body condition. That is, hibernating to avoid predation forces squirrels to risk starvation by allocating excess energetic reserves to prolonging hibernation via early immergence when squirrels could otherwise easily survive winter on purely energetic terms. Hence, hibernators must navigate an annual variant of the fundamental ecological trade‐off between predation risk and energetic acquisition that shapes the evolution of optimal annual routines (McNamara & Houston, 2008; Werner & Anholt, 1993).

We modelled seasonal body mass changes of northern Idaho ground squirrels—a federally threatened hibernator—based on >3000 records that incorporated intrinsic and environmental covariates. In doing so, we sought to better understand how seasonality interacts with multiple selection pressures to influence optimal life‐history strategies in animals faced with allocation trade‐offs that affect demography. Northern Idaho ground squirrels exhibited a remarkable ability to rapidly gain mass through the deposition of endogenous fat stores, which allowed the squirrels to survive hibernation periods of up to 300 days. This extensive annual hibernation encompasses not only the harsh montane winters of the region but also the second half of summer, when food is readily available. Thus, our results demonstrate that these squirrels may rarely—if ever—be at risk of failing to acquire sufficient stores to survive the annual period of food shortage per se. Squirrels in relatively good condition appear to allocate excess energetic reserves to improving overwinter survival probability, likely by prolonging hibernation to avoid predation. Cool active‐season temperatures and possibly low conspecific density—conditions associated with decreased foraging activity—constrained body condition, and hence, resource allocation and demographic outcomes. Overall, we provide a comprehensive mechanistic perspective on the seasonal body mass dynamics of these hibernators that helps explain how food seasonality influences adaptive life‐history optimization. The seasonal body mass curves we constructed may also be a useful tool for evaluating the effectiveness of conservation actions aimed at benefitting northern Idaho ground squirrels. Specifically, model‐based predictions of body mass across the active season provide a baseline expectation against which managers can compare empirical squirrel body mass records to assess relative body condition. Moreover, our survival estimates pinpoint a time—shortly prior to the onset of hibernation at a given site—when such comparisons may be most useful as an indicator of short‐term future population dynamics. Further research testing the mechanisms suggested by our results may provide insights beneficial to managers who seek to promote the persistence of northern Idaho ground squirrels and other hibernators in our rapidly changing world.

## AUTHOR CONTRIBUTIONS

Austin Z. T. Allison, Courtney J. Conway and Amanda R. Goldberg conceived and designed the study. Austin Z. T. Allison, Amanda R. Goldberg, Alice E. Morris and Emma C. Hakanson collected and curated the data. Austin Z. T. Allison and Courtney J. Conway analysed the data. Austin Z. T. Allison led the writing of the manuscript. All authors contributed critically to revising the manuscript.

## CONFLICT OF INTEREST STATEMENT

The authors declare no conflict of interest in relation to the content or publication of this manuscript.

## Supporting information


**Table S1.** Site‐level mean values (±SD) for annual environmental predictor variables considered in linear mixed‐effects models to explain variation in northern Idaho ground squirrel body mass. Mean and standard deviations are based on years during which a given study site was included in the study. Not all sites were trapped in all years of the study and squirrel body mass records were unevenly distributed among sites and years.
**Table S2.** Variables that explained intraspecific variation in northern Idaho ground squirrel body mass based on 8753 body mass records from 3351 squirrels. The table includes all candidate linear mixed‐effects models that received ≥0.01 Akaike model weight (*w*_*i*_), the global model (bolded) and the random intercept‐only model (italicized). Random intercepts are included in parentheses. Variable notation is as follows: JDay = day of year, Sex = squirrel sex, Age = squirrel age class, Melt.Avg = mean snowmelt date, Rel.Melt = annual snowmelt date relative to the site mean, Rel.Temp = annual active‐season temperature relative to the site mean, NDVI = normalized difference vegetation index, NIDGS = northern Idaho ground squirrel density, COGS = Columbian ground squirrel density, ID = individual squirrel ID, Site = study site, Year = year.
**Table S3.** Variables that explained intraspecific variation in northern Idaho ground squirrel overwinter recapture probability (i.e. survival probability) based on 2467 overwinter return intervals for 1856 squirrels. The table includes all candidate generalized linear mixed‐effects models that received ≥0.01 Akaike model weight (*w*_*i*_), the global model (bolded) and the random intercept‐only model (italicized). Random intercepts are included in parentheses. Variable notation is as follows: Sex = squirrel sex, Age = squirrel age class, Condition = pre‐hibernation body condition, ID = individual squirrel ID, Site = study site, Year = year.
**Table S4.** Beta estimates for fixed effects terms in the top linear mixed‐effects model to explain intraspecific variation in northern Idaho ground squirrel body mass based on 8753 body mass records from 3351 squirrels. Factor levels denote the polynomial degree and/or categorical level of a given effect (e.g. the effect of being male compared to a female baseline). The top model also included crossed random intercepts for individual squirrel ID, study site and year.
**Table S5.** Beta estimates for fixed effects terms in the second‐ranked linear mixed‐effects model to explain intraspecific variation in northern Idaho ground squirrel body mass based on 8753 body mass records from 3351 squirrels. Factor levels denote the polynomial degree and/or categorical level of a given effect (e.g. the effect of being male compared to a female baseline). The model also included crossed random intercepts for individual squirrel ID, study site and year. The model contains the same model structure as the top model plus an additive effect of NDVI. NDVI is an uninformative predictor variable because its addition to the top model reduces model fit and the term is non‐significant while parameter estimates for the other terms remain nearly identical as in the top model.
**Table S6.** Beta estimates for fixed effects terms in the third‐ranked linear mixed‐effects model to explain intraspecific variation in northern Idaho ground squirrel body mass based on 8753 body mass records from 3351 squirrels. Factor levels denote the polynomial degree and/or categorical level of a given effect (e.g. the effect of being male compared to a female baseline). The model also included crossed random intercepts for individual squirrel ID, study site and year. The model contains the same model structure as the top model plus an additive effect of Columbian ground squirrel density. Columbian ground squirrel density is an uninformative predictor variable because its addition to the top model reduces model fit and the term is non‐significant while parameter estimates for the other terms remain nearly identical as in the top model.
**Table S7.** Beta estimates for fixed effects terms in the fourth‐ranked linear mixed‐effects model to explain intraspecific variation in northern Idaho ground squirrel body mass based on 8753 body mass records from 3351 squirrels. Factor levels denote the polynomial degree and/or categorical level of a given effect (e.g. the effect of being male compared to a female baseline). The model also included crossed random intercepts for individual squirrel ID, study site and year. The model contains the same model structure as the top model plus an additive effect of relative snowmelt date. Interannual snowmelt date is an uninformative predictor variable because its addition to the top model reduces model fit and the term is non‐significant while parameter estimates for the other terms remain nearly identical as in the top model.
**Table S8.** Beta estimates for fixed effects terms in the top generalized linear mixed‐effects model to explain variation in northern Idaho ground squirrel overwinter recapture probability (i.e. survival probability) based on 2467 overwinter return intervals for 1856 squirrels. Factor levels denote the categorical level of a given effect (e.g. the effect of being male compared to a female baseline). The top model also included crossed random intercepts for individual squirrel ID, study site and year.

## Data Availability

Data available from the Figshare Digital Repository: https://doi.org/10.6084/m9.figshare.28821986 (Allison et al., 2025).
