# Supplementary material for: Seasonal body mass dynamics mediate life‐history trade‐offs in a hibernating mammal
Source: J Anim Ecol. 2025 Oct 18;95(3):383–96. doi: 10.1111/1365-2656.70160 (PMC12957731; doi:10.1111/1365-2656.70160)
Supplement: Supplementary file 1 — Table S1. Site‐level mean values (±SD) for annual environmental predictor variables considered in linear mixed‐effects models to explain variation in northern Idaho ground squirrel body mass. Mean and standard deviations are based on years during which a given study site was included in the study. Not all sites were trapped in all years of the study and squirrel body mass records were unevenly distributed among sites and years. Table S2. Variables that explained intraspecific variation in northern Idaho ground squirrel body mass based on 8753 body mass records from 3351 squirrels. The table includes all candidate linear mixed‐effects models that received ≥0.01 Akaike model weight (w i ), the global model (bolded) and the random intercept‐only model (italicized). Random intercepts are included in parentheses. Variable notation is as follows: JDay = day of year, Sex = squirrel sex, Age = squirrel age class, Melt.Avg = mean snowmelt date, Rel.Melt = annual snowmelt date relative to the site mean, Rel.Temp = annual active‐season temperature relative to the site mean, NDVI = normalized difference vegetation index, NIDGS = northern Idaho ground squirrel density, COGS = Columbian ground squirrel density, ID = individual squirrel ID, Site = study site, Year = year. Table S3. Variables that explained intraspecific variation in northern Idaho ground squirrel overwinter recapture probability (i.e. survival probability) based on 2467 overwinter return intervals for 1856 squirrels. The table includes all candidate generalized linear mixed‐effects models that received ≥0.01 Akaike model weight (w i ), the global model (bolded) and the random intercept‐only model (italicized). Random intercepts are included in parentheses. Variable notation is as follows: Sex = squirrel sex, Age = squirrel age class, Condition = pre‐hibernation body condition, ID = individual squirrel ID, Site = study site, Year = year. Table S4. Beta estimates for fixed effects terms in the top linear mixed‐effec [file JANE-95-383-s001.docx]

**Table S1.** Site-level mean values (±SD) for annual environmental predictor variables considered in linear mixed-effects models to explain variation in northern Idaho ground squirrel body mass. Means and standard deviations are based on years during which a given study site was included in the study. Not all sites were trapped in all years of the study and squirrel body mass records were unevenly distributed among sites and years.

| **Study Site** | **Elevation (m)** | **Snowmelt Timing (Day of Year)** | **Active-Season Temperature (°C)** | **Active-Season NDVI** | **NIDGS Density (squirrels/ha)** | **COGS Density (squirrels/ha)** |
| --- | --- | --- | --- | --- | --- | --- |
| Mud Creek | 1229 | 71.5 ± 9.5 | 24.2 ± 0.6 | 0.62 ± 0.07 | 14.7 ± 5.4 | 1.8 ± 2.0 |
| Tamarack East | 1275 | 88.8 ± 16.9 | 23.7 ± 1.1 | 0.43 ± 0.03 | 4.8 ± 3.7 | 1.2 ± 1.0 |
| Summit Gulch | 1328 | 75.5 ± 11.2 | 22.5 ± 1.1 | 0.52 ± 0.04 | 1.1 ± 1.2 | 0.0 ± 0.0 |
| Squirrel Manor | 1348 | 68.5 ± 6.0 | 23.2 ± 0.6 | 0.46 ± 0.07 | 16.7 ± 4.7 | 0.0 ± 0.0 |
| Squirrel Valley | 1348 | 77.0 ± 16.6 | 23.2 ± 0.6 | 0.42 ± 0.05 | 28.9 ± 3.7 | 0.0 ± 0.0 |
| Steve's Creek | 1348 | 85.2 ± 16.8 | 22.8 ± 1.1 | 0.43 ± 0.05 | 9.9 ± 6.3 | 4.5 ± 2.0 |
| Fawn Creek | 1367 | 92.3 ± 16.1 | 22.3 ± 1.2 | 0.49 ± 0.06 | 4.6 ± 1.5 | 2.8 ± 1.7 |
| Cap Gun | 1383 | 76.2 ± 13.5 | 23.1 ± 1.2 | 0.53 ± 0.03 | 0.5 ± 0.2 | 1.9 ± 1.6 |
| Lost Valley | 1487 | 92.5 ± 21.1 | 22.1 ± 1.2 | 0.50 ± 0.02 | 1.5 ± 0.4 | 10.5 ± 4.9 |
| Huckleberry | 1493 | 109.0 ± 20.2 | 21.0 ± 1.1 | 0.49 ± 0.03 | 0.4 ± 0.6 | 4.2 ± 3.2 |
| Slaughter Gulch | 1495 | 97.8 ± 18.0 | 22.2 ± 1.2 | 0.36 ± 0.05 | 1.7 ± 0.9 | 2.2 ± 1.2 |
| Cold Springs West | 1561 | 119.8 ± 10.7 | 20.7 ± 1.1 | 0.50 ± 0.03 | 0.7 ± 0.5 | 6.8 ± 2.9 |
| Cold Springs East | 1582 | 122.5 ± 11.4 | 20.8 ± 1.1 | 0.49 ± 0.04 | 2.9 ± 0.9 | 0.0 ± 0.1 |
| YCC | 1596 | 94.0 ± 16.3 | 22.1 ± 1.1 | 0.40 ± 0.03 | 1.5 ± 1.4 | 5.7 ± 2.9 |
| Lower Butter 2 | 1600 | 113.8 ± 20.7 | 21.1 ± 1.2 | 0.40 ± 0.04 | 4.8 ± 2.7 | 1.2 ± 0.8 |
| Rocky Top | 1702 | 112 ± 16.6 | 22.1 ± 1.1 | 0.41 ± 0.02 | 4.8 ± 1.8 | 0.6 ± 0.5 |

**Table S2.** Variables that explained intraspecific variation in northern Idaho ground squirrel body mass based on 8753 body mass records from 3351 squirrels. The table includes all candidate linear mixed-effects models that received ≥0.01 Akaike model weight (w_i_), the global model (bolded), and the random intercept-only model (italicized). Random intercepts are included in parentheses. Variable notation is as follows: JDay = day of year, Sex = squirrel sex, Age = squirrel age class, Melt.Avg = mean snowmelt date, Rel.Melt = annual snowmelt date relative to the site mean, Rel.Temp = annual active-season temperature relative to the site mean, NDVI = normalized difference vegetation index, NIDGS = northern Idaho ground squirrel density, COGS = Columbian ground squirrel density, ID = individual squirrel ID, Site = study site, Year = year.

| **Model** | **ΔAIC_c_** | ***w_i_*** |
| --- | --- | --- |
| JDay^3^ × Sex × Age + Melt.Avg + Rel.Temp + NIDGS + (ID) + (Site) + (Year) | 0.00 | 0.37 |
| JDay^3^ × Sex × Age + Melt.Avg + Rel.Temp + NDVI + NIDGS + (ID) + (Site) + (Year) | 1.69 | 0.16 |
| JDay^3^ × Sex × Age + Melt.Avg + Rel.Temp + NIDGS + COGS + (ID) + (Site) + (Year) | 2.01 | 0.14 |
| JDay^3^ × Sex × Age + Melt.Avg + Rel.Melt + Rel.Temp + NIDGS + (ID) + (Site) + (Year) | 2.01 | 0.14 |
| JDay^3^ × Sex × Age + Melt.Avg + Rel.Temp + NDVI + NIDGS + COGS + (ID) + (Site) + (Year) | 3.70 | 0.06 |
| JDay^3^ × Sex × Age + Melt.Avg + Rel.Melt + Rel.Temp + NDVI + NIDGS + (ID) + (Site) + (Year) | 3.70 | 0.06 |
| JDay^3^ × Sex × Age + Melt.Avg + Rel.Melt + Rel.Temp + NIDGS + COGS + (ID) + (Site) + (Year) | 4.02 | 0.05 |
| **JDay^3^ × Sex × Age + Melt.Avg + Rel.Melt + Rel.Temp + NDVI + NIDGS + COGS + (ID) + (Site) + (Year)** | 5.72 | 0.02 |
| *(ID) + (Site) + (Year)* | 12704.27 | 0.00 |

**Table S3.** Variables that explained intraspecific variation in northern Idaho ground squirrel overwinter recapture probability (i.e., survival probability) based on 2467 overwinter return intervals for 1856 squirrels. The table includes all candidate generalized linear mixed-effects models that received ≥0.01 Akaike model weight (w_i_), the global model (bolded), and the random intercept-only model (italicized). Random intercepts are included in parentheses. Variable notation is as follows: Sex = squirrel sex, Age = squirrel age class, Condition = pre-hibernation body condition, ID = individual squirrel ID, Site = study site, Year = year.

| **Model** | **ΔAIC_c_** | ***w_i_*** |
| --- | --- | --- |
| **Sex + Age + Condition + (ID) + (Site) + (Year)** | 0.00 | 0.99 |
| Sex + Age + (ID) + (Site) + (Year) | 8.69 | 0.01 |
| *(ID) + (Site) + (Year)* | 116.07 | 0.00 |

**Table S4.** Beta estimates for fixed effects terms in the top linear mixed-effects model to explain intraspecific variation in northern Idaho ground squirrel body mass based on 8753 body mass records from 3351 squirrels. Factor levels denote the polynomial degree and/or categorical level of a given effect (e.g., the effect of being male compared to a female baseline). The top model also included crossed random intercepts for individual squirrel ID, study site, and year.

| **Model Term** | **Factor Level** | **Estimated *β* Coefficient** | **Standard Error** | ***t*-value** |
| --- | --- | --- | --- | --- |
| Day of Year | 1^st^ degree polynomial | 40.25 | 1.26 | 31.97 |
| Day of Year | 2^nd^ degree polynomial | 23.91 | 1.46 | 16.43 |
| Day of Year | 3^rd^ degree polynomial | 8.48 | 1.26 | 6.74 |
| Sex | Male | 1.34 | 0.03 | 39.02 |
| Age Class | Juvenile | -2.29 | 0.17 | -13.64 |
| Age Class | Yearling | -0.33 | 0.02 | -19.96 |
| Day of Year × Sex | 1^st^ degree polynomial, Male | 78.37 | 3.54 | 22.12 |
| Day of Year × Sex | 2^nd^ degree polynomial, Male | 2.02 | 4.30 | 0.47 |
| Day of Year × Sex | 3^rd^ degree polynomial, Male | -8.98 | 2.61 | -3.43 |
| Day of Year × Age Class | 1^st^ degree polynomial, Juvenile | 45.50 | 20.29 | 2.24 |
| Day of Year × Age Class | 2^nd^ degree polynomial, Juvenile | -16.51 | 11.68 | -1.41 |
| Day of Year × Age Class | 3^rd^ degree polynomial, Juvenile | -9.53 | 4.35 | -2.19 |
| Day of Year × Age Class | 1^st^ degree polynomial, Yearling | 4.27 | 1.64 | 2.60 |
| Day of Year × Age Class | 2^nd^ degree polynomial, Yearling | -7.74 | 1.92 | -4.03 |
| Day of Year × Age Class | 3^rd^ degree polynomial, Yearling | -3.24 | 1.71 | -1.90 |
| Sex × Age Class | Male, Juvenile | -1.46 | 0.24 | -6.16 |
| Sex × Age Class | Male, Yearling | -0.55 | 0.03 | -16.41 |
| Day of Year × Sex × Age Class | 1^st^ degree polynomial, Male, Juvenile | -56.01 | 28.72 | -1.95 |
| Day of Year × Sex × Age Class | 2^nd^ degree polynomial, Male, Juvenile | -15.09 | 17.01 | -0.89 |
| Day of Year × Sex × Age Class | 3^rd^ degree polynomial, Male, Juvenile | 14.55 | 6.72 | 2.16 |
| Day of Year × Sex × Age Class | 1^st^ degree polynomial, Male, Yearling | -28.96 | 3.86 | -7.51 |
| Day of Year × Sex × Age Class | 2^nd^ degree polynomial, Male, Yearling | -11.06 | 4.66 | -2.37 |
| Day of Year × Sex × Age Class | 3^rd^ degree polynomial, Male, Yearling | -1.03 | 3.14 | -0.33 |
| Mean Snowmelt Date |  | -0.24 | 0.03 | -7.87 |
| Interannual Temperature |  | 0.16 | 0.04 | 4.32 |
| Conspecific Density |  | 0.08 | 0.02 | 5.17 |

**Table S5.** Beta estimates for fixed effects terms in the second-ranked linear mixed-effects model to explain intraspecific variation in northern Idaho ground squirrel body mass based on 8753 body mass records from 3351 squirrels. Factor levels denote the polynomial degree and/or categorical level of a given effect (e.g., the effect of being male compared to a female baseline). The model also included crossed random intercepts for individual squirrel ID, study site, and year. The model contains the same model structure as the top model plus an additive effect of NDVI. NDVI is an uninformative predictor variable because its addition to the top model reduces model fit and the term is non-significant while parameter estimates for the other terms remain nearly identical as in the top model.

| **Model Term** | **Factor Level** | **Estimated *β* Coefficient** | **Standard Error** | ***t*-value** |
| --- | --- | --- | --- | --- |
| Day of Year | 1^st^ degree polynomial | 40.22 | 1.26 | 31.93 |
| Day of Year | 2^nd^ degree polynomial | 23.91 | 1.46 | 16.42 |
| Day of Year | 3^rd^ degree polynomial | 8.45 | 1.26 | 6.71 |
| Sex | Male | 1.34 | 0.03 | 39.03 |
| Age Class | Juvenile | -2.30 | 0.17 | -13.65 |
| Age Class | Yearling | -0.33 | 0.02 | -19.87 |
| Day of Year × Sex | 1^st^ degree polynomial, Male | 78.42 | 3.54 | 22.13 |
| Day of Year × Sex | 2^nd^ degree polynomial, Male | 2.07 | 4.30 | 0.48 |
| Day of Year × Sex | 3^rd^ degree polynomial, Male | -8.93 | 2.61 | -3.42 |
| Day of Year × Age Class | 1^st^ degree polynomial, Juvenile | 45.90 | 20.30 | 2.26 |
| Day of Year × Age Class | 2^nd^ degree polynomial, Juvenile | -16.69 | 11.68 | -1.43 |
| Day of Year × Age Class | 3^rd^ degree polynomial, Juvenile | -9.45 | 4.35 | -2.17 |
| Day of Year × Age Class | 1^st^ degree polynomial, Yearling | 4.29 | 1.64 | 2.61 |
| Day of Year × Age Class | 2^nd^ degree polynomial, Yearling | -7.74 | 1.92 | -4.03 |
| Day of Year × Age Class | 3^rd^ degree polynomial, Yearling | -3.23 | 1.71 | -1.89 |
| Sex × Age Class | Male, Juvenile | -1.46 | 0.24 | -6.16 |
| Sex × Age Class | Male, Yearling | -0.55 | 0.03 | -16.42 |
| Day of Year × Sex × Age Class | 1^st^ degree polynomial, Male, Juvenile | -56.15 | 28.72 | -1.96 |
| Day of Year × Sex × Age Class | 2^nd^ degree polynomial, Male, Juvenile | -15.09 | 17.01 | -0.89 |
| Day of Year × Sex × Age Class | 3^rd^ degree polynomial, Male, Juvenile | 14.48 | 6.73 | 2.15 |
| Day of Year × Sex × Age Class | 1^st^ degree polynomial, Male, Yearling | -29.00 | 3.86 | -7.51 |
| Day of Year × Sex × Age Class | 2^nd^ degree polynomial, Male, Yearling | -11.10 | 4.66 | -2.38 |
| Day of Year × Sex × Age Class | 3^rd^ degree polynomial, Male, Yearling | -1.06 | 3.14 | -0.34 |
| Mean Snowmelt Date |  | -0.23 | 0.03 | -7.72 |
| Interannual Temperature |  | 0.16 | 0.04 | 4.33 |
| Conspecific Density |  | 0.08 | 0.02 | 5.19 |
| NDVI |  | 0.01 | 0.01 | 0.57 |

**Table S6.** Beta estimates for fixed effects terms in the third-ranked linear mixed-effects model to explain intraspecific variation in northern Idaho ground squirrel body mass based on 8753 body mass records from 3351 squirrels. Factor levels denote the polynomial degree and/or categorical level of a given effect (e.g., the effect of being male compared to a female baseline). The model also included crossed random intercepts for individual squirrel ID, study site, and year. The model contains the same model structure as the top model plus an additive effect of Columbian ground squirrel density. Columbian ground squirrel density is an uninformative predictor variable because its addition to the top model reduces model fit and the term is non-significant while parameter estimates for the other terms remain nearly identical as in the top model.

| **Model Term** | **Factor Level** | **Estimated *β* Coefficient** | **Standard Error** | ***t*-value** |
| --- | --- | --- | --- | --- |
| Day of Year | 1^st^ degree polynomial | 40.25 | 1.26 | 31.96 |
| Day of Year | 2^nd^ degree polynomial | 23.91 | 1.46 | 16.42 |
| Day of Year | 3^rd^ degree polynomial | 8.47 | 1.26 | 6.73 |
| Sex | Male | 1.34 | 0.03 | 39.02 |
| Age Class | Juvenile | -2.29 | 0.17 | -13.63 |
| Age Class | Yearling | -0.33 | 0.02 | -19.95 |
| Day of Year × Sex | 1^st^ degree polynomial, Male | 78.38 | 3.54 | 22.12 |
| Day of Year × Sex | 2^nd^ degree polynomial, Male | 2.03 | 4.30 | 0.47 |
| Day of Year × Sex | 3^rd^ degree polynomial, Male | -8.95 | 2.61 | -3.42 |
| Day of Year × Age Class | 1^st^ degree polynomial, Juvenile | 45.38 | 20.3 | 2.24 |
| Day of Year × Age Class | 2^nd^ degree polynomial, Juvenile | -16.44 | 11.68 | -1.41 |
| Day of Year × Age Class | 3^rd^ degree polynomial, Juvenile | -9.55 | 4.35 | -2.19 |
| Day of Year × Age Class | 1^st^ degree polynomial, Yearling | 4.26 | 1.64 | 2.60 |
| Day of Year × Age Class | 2^nd^ degree polynomial, Yearling | -7.73 | 1.92 | -4.03 |
| Day of Year × Age Class | 3^rd^ degree polynomial, Yearling | -3.24 | 1.71 | -1.90 |
| Sex × Age Class | Male, Juvenile | -1.46 | 0.24 | -6.17 |
| Sex × Age Class | Male, Yearling | -0.55 | 0.03 | -16.41 |
| Day of Year × Sex × Age Class | 1^st^ degree polynomial, Male, Juvenile | -55.87 | 28.73 | -1.95 |
| Day of Year × Sex × Age Class | 2^nd^ degree polynomial, Male, Juvenile | -15.20 | 17.02 | -0.89 |
| Day of Year × Sex × Age Class | 3^rd^ degree polynomial, Male, Juvenile | 14.56 | 6.73 | 2.17 |
| Day of Year × Sex × Age Class | 1^st^ degree polynomial, Male, Yearling | -28.98 | 3.86 | -7.51 |
| Day of Year × Sex × Age Class | 2^nd^ degree polynomial, Male, Yearling | -11.08 | 4.66 | -2.38 |
| Day of Year × Sex × Age Class | 3^rd^ degree polynomial, Male, Yearling | -1.05 | 3.14 | -0.34 |
| Mean Snowmelt Date |  | -0.24 | 0.03 | -7.65 |
| Interannual Temperature |  | 0.16 | 0.04 | 4.25 |
| Conspecific Density |  | 0.08 | 0.02 | 5.13 |
| Competitor Density |  | 0.00 | 0.01 | 0.25 |

**Table S7.** Beta estimates for fixed effects terms in the fourth-ranked linear mixed-effects model to explain intraspecific variation in northern Idaho ground squirrel body mass based on 8753 body mass records from 3351 squirrels. Factor levels denote the polynomial degree and/or categorical level of a given effect (e.g., the effect of being male compared to a female baseline). The model also included crossed random intercepts for individual squirrel ID, study site, and year. The model contains the same model structure as the top model plus an additive effect of relative snowmelt date. Interannual snowmelt date is an uninformative predictor variable because its addition to the top model reduces model fit and the term is non-significant while parameter estimates for the other terms remain nearly identical as in the top model.

| **Model Term** | **Factor Level** | **Estimated *β* Coefficient** | **Standard Error** | ***t*-value** |
| --- | --- | --- | --- | --- |
| Day of Year | 1^st^ degree polynomial | 40.25 | 1.26 | 31.96 |
| Day of Year | 2^nd^ degree polynomial | 23.91 | 1.46 | 16.43 |
| Day of Year | 3^rd^ degree polynomial | 8.47 | 1.26 | 6.74 |
| Sex | Male | 1.34 | 0.03 | 39.01 |
| Age Class | Juvenile | -2.30 | 0.17 | -13.64 |
| Age Class | Yearling | -0.33 | 0.02 | -19.93 |
| Day of Year × Sex | 1^st^ degree polynomial, Male | 78.37 | 3.54 | 22.12 |
| Day of Year × Sex | 2^nd^ degree polynomial, Male | 2.02 | 4.30 | 0.47 |
| Day of Year × Sex | 3^rd^ degree polynomial, Male | -8.98 | 2.61 | -3.43 |
| Day of Year × Age Class | 1^st^ degree polynomial, Juvenile | 45.50 | 20.29 | 2.24 |
| Day of Year × Age Class | 2^nd^ degree polynomial, Juvenile | -16.51 | 11.68 | -1.41 |
| Day of Year × Age Class | 3^rd^ degree polynomial, Juvenile | -9.53 | 4.35 | -2.19 |
| Day of Year × Age Class | 1^st^ degree polynomial, Yearling | 4.27 | 1.64 | 2.60 |
| Day of Year × Age Class | 2^nd^ degree polynomial, Yearling | -7.74 | 1.92 | -4.03 |
| Day of Year × Age Class | 3^rd^ degree polynomial, Yearling | -3.24 | 1.71 | -1.89 |
| Sex × Age Class | Male, Juvenile | -1.46 | 0.24 | -6.16 |
| Sex × Age Class | Male, Yearling | -0.55 | 0.03 | -16.4 |
| Day of Year × Sex × Age Class | 1^st^ degree polynomial, Male, Juvenile | -56.02 | 28.72 | -1.95 |
| Day of Year × Sex × Age Class | 2^nd^ degree polynomial, Male, Juvenile | -15.09 | 17.01 | -0.89 |
| Day of Year × Sex × Age Class | 3^rd^ degree polynomial, Male, Juvenile | 14.55 | 6.73 | 2.16 |
| Day of Year × Sex × Age Class | 1^st^ degree polynomial, Male, Yearling | -28.97 | 3.86 | -7.50 |
| Day of Year × Sex × Age Class | 2^nd^ degree polynomial, Male, Yearling | -11.06 | 4.66 | -2.37 |
| Day of Year × Sex × Age Class | 3^rd^ degree polynomial, Male, Yearling | -1.03 | 3.14 | -0.33 |
| Mean Snowmelt Date |  | -0.24 | 0.03 | -7.85 |
| Interannual Temperature |  | 0.16 | 0.04 | 4.29 |
| Conspecific Density |  | 0.08 | 0.02 | 5.15 |
| Interannual Snowmelt Date |  | 0.00 | 0.00 | -0.03 |

**Table S8.** Beta estimates for fixed effects terms in the top generalized linear mixed-effects model to explain variation in northern Idaho ground squirrel overwinter recapture probability (i.e., survival probability) based on 2467 overwinter return intervals for 1856 squirrels. Factor levels denote the categorical level of a given effect (e.g., the effect of being male compared to a female baseline). The top model also included crossed random intercepts for individual squirrel ID, study site, and year.

| **Model Term** | **Factor Level** | **Estimated *β* Coefficient** | **Standard Error** | ***z*-value** |
| --- | --- | --- | --- | --- |
| Sex | Male | -0.70 | 0.10 | -7.19 |
| Age Class | Juvenile | -0.84 | 0.12 | -6.77 |
| Age Class | Yearling | -0.23 | 0.12 | -1.95 |
| Body Condition |  | 0.17 | 0.05 | 3.27 |
